# AA amyloid in human food chain is a possible biohazard

**DOI:** 10.1038/s41598-021-00588-w

**Published:** 2021-10-26

**Authors:** Anna Rising, Paola Gherardi, Gefei Chen, Jan Johansson, Marie E. Oskarsson, Gunilla T. Westermark, Per Westermark

**Affiliations:** 1 Department of Biosciences and Nutrition, Karolinska Institutet, Huddinge, Huddinge, Sweden; 2 Department of Anatomy, Physiology and Biochemistry, Swedish University of Agricultural Sciences, Uppsala, Sweden; 3 Unità Operativa Igiene Degli Alimenti Di Origine Animale, Azienda Unità Sanitaria Locale Di Piacenza, Piazzale Milano, Piacenza, Italy; 4 Department of Medical Cell Biology, Uppsala University, Uppsala, Sweden; 5 Rudbeck Laboratory, C11, Department of Immunology, Genetics and Pathology, Uppsala University, 75185 Uppsala, Sweden

**Keywords:** Biochemistry, Neuroscience, Pathogenesis, Risk factors

## Abstract

AA amyloidosis can be transmitted experimentally in several mammalian and avian species as well as spontaneously between captive animals, even by oral intake of amyloid seeds. Amyloid seeding can cross species boundaries, and fibrils of one kind of amyloid protein may also seed other types. Here we show that meat from Swedish and Italian cattle for consumption by humans often contains AA amyloid and that bovine AA fibrils efficiently cross-seed human amyloid β peptide, associated with Alzheimer’s disease.

## Introduction

Human AA amyloidosis is one of the most prevalent systemic amyloidoses worldwide. The amyloid fibrils are built up by protein AA, derived from the 104-residue acute phase reactant serum AA (SAA). Persistently high plasma concentration of SAA is a prerequisite for the AA amyloidosis development, why the condition often follows severe chronic inflammatory disorders^1–3^. AA amyloidosis is also frequent in many mammalian and avian species, both wild-living and domesticated^4^. In mammals, including human and bovine, AA amyloid deposits develop in most organs of the body through seeding where misfolded SAA species catalyze fibril formation leading to large extracellular aggregates, i.e. amyloid^5^. An earlier report indicated that AA amyloidosis might occur in Japanese cattle^6^ which made us question whether this is seen in European cattle used for human consumption.

## Results and discussion

**Figure 1:**
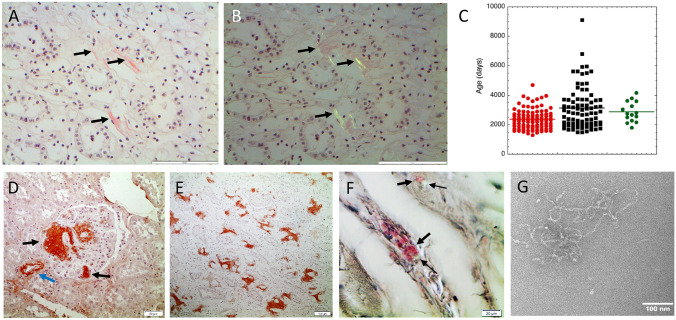
(**A**,**B**) A section of kidney from Swedish cattle # 9 with sparse amyloid (arrows) in outer medulla (bar = 50 μm). The section was stained with Congo red and viewed in normal light in (**A**) and in polarized light between crossed polars in (**B**). (**C**) Age of individual animals in the Swedish (red) and Italian (black) materials. Italian cows with amyloid are in green. (**D**) Italian bovine kidney with amyloid deposits in the glomerulus (black arrow) and arteriole (blue arrow), immunolabeled with anti protein AA antibodies. (**E**) Large amount of immunolabeled AA amyloid in the renal medulla. (**F**) Amyloid (arrows) in striated muscle, stained with Congo red and examined between partially crossed polarizers. A faint green birefringence is seen (thin arrows). (**G**) Fibrils from purified bovine protein AA, negatively contrasted with uranyl acetate. Bar (**A**,**B**) 100 μm, (**D**) 50 μm, (**E**) 100 μm, (**F**) 20 μm, (**G**) 100 nm. All sections (except **G**) were counter-stained with hematoxylin.

**Figure 2:**
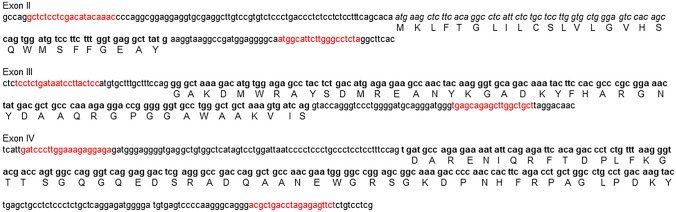
The nucleotide sequence of exons II–IV coding for bovine serum amyloid A (SAA) (NC_037356) with 5’ and 3’ flanking regions of the introns, presented in lowercase. The signal peptide of SAA is in italic, the mature SAA peptide in bold, and the primer binding sequences are indicated in red. Uppercase letters correspond to the one-letter code of the SAA amino acid sequence.

Tissues for histopathological examination were obtained from 97 Swedish cattle > 4 years old from one local slaughterhouse. All animals but one were found healthy at slaughter and used for food production. In one of the animals, age 11 years and judged healthy, AA amyloid was found in sections from the kidney (Fig. 1A,B). We also obtained tissue material from 99 Italian cattle from a single slaughterhouse. These animals were also > 4 years old but on average significantly older than the Swedish animals (*p* = 0.0004; Fig. 1C). All were deemed healthy except two that had lesions: one had a parasite infection and the other liver abscesses. Out of the 99 Italian cattle, 15 had AA amyloid deposits, including one of the two animals with post mortem remarks. All of the Italian amyloidotic animals had renal deposits (Fig. 1D,E). Three of the 15 animals had amyloid deposits in the striated muscle samples (Fig. 1F). Surprisingly, none of the 12 oldest, of which one was almost 25 years old, had amyloidosis. The reason for the high amyloidosis prevalence in Italian versus the Swedish animals is unclear and the found values may not be representative of the prevalence on a national level since the material came from only one region in each country. However, there may be differences between the two groups of animals in terms of the history of disease or housing and management. A potential mutation in the SAA gene in the Italian cattle, causing the AA protein to be more aggregation-prone could be another plausible explanation. However, the SAA protein sequences obtained from Italian and Swedish animals were identical as determined by gene exon sequencing (Fig. 2).

**Figure 3:**
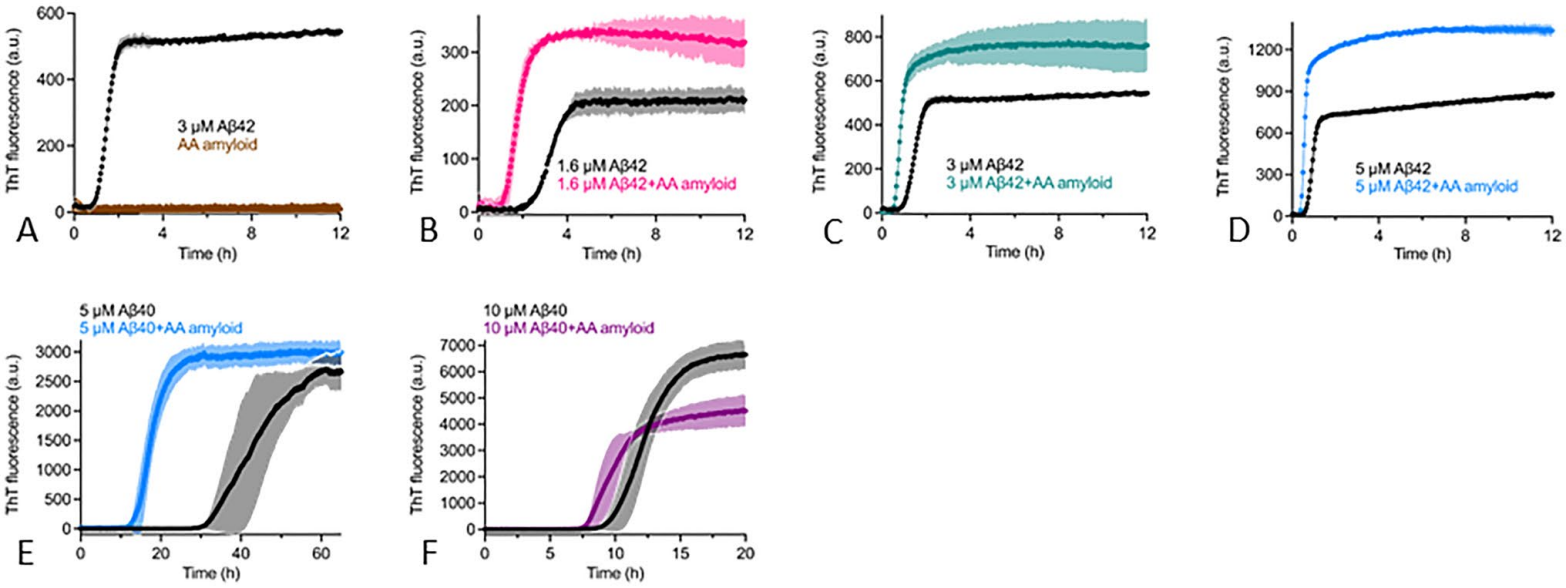
(**A**–**D**). AA amyloid from cattle cross seeds human Aβ42 in vitro. (**A**) 3 µM recombinant Aβ42 and AA amyloid isolated from a bovine kidney (400 × dilution from the stock as final concentration) were monitored separately in the presence of ThT at 37 °C. Aggregation traces of different concentrations of Aβ42, i.e., 1.6 µM (**B**), 3 µM (**C**) and 5 µM (**D**) with and without bovine AA amyloid (400 × dilution from the stock as final concentration) at 37 °C. The data is presented as mean with error bars (SD from four replicates). (**E**,**F**) AA amyloid fibrils from cattle cross seed human Aβ40 in vitro. Aggregation traces of different concentrations of Aβ40, *i.e.,* 5 µM (**E**) and 10 µM (**F**) with and without AA amyloid (400 × dilution from the stock as final concentration) at 37 °C. The data is presented as mean with error bars (SD from four replicates).

Next question was whether consumption of AA amyloid could constitute a risk factor for human disease. Spreading of amyloid in the organism as well as between individuals occurs through seeding. Transmission of AA amyloidosis between individual animals has been shown to occur experimentally in mice^7^ and spontaneously in captive cheetahs^8^, even via the oral route by intake of seeds. Cross-seeding, by which aggregates of one kind can induce amyloid formation of a similar or different protein molecule and between different mammalian species, was evident in the outbreak of variant Creutzfeldt Jakob disease when susceptible individuals developed the illness after ingesting contaminated meat from cattle with bovine spongiform encephalitis^9^. Moreover, iatrogenic induced seeding of the prion protein and amyloid β peptide (Aβ) in the brain has been observed in patients treated with pituitary gland extracts^10^. Aβ is the main constituent of the fibril plaques that are characteristic for Alzheimer’s disease and likely a major player in the pathogenesis of this disease^11^. Since seeding has been implicated for several amyloid disorders, including Parkinson’s and Alzheimer’s diseases^12^, we questioned if bovine AA protein fibrils could cross-seed human Aβ, in particular since we unexpectedly found that one ‘gold standard’ anti-human Aβ mouse monoclonal antibody 4G8 binds to human protein AA, indicating conformational similarities that may promote intermolecular interactions (see Supplementary Information). To study this, bovine amyloid protein AA fibrils (Fig. 1G) (see Supplementary Information) and human Aβ42 and Aβ40 were used. First, we monitored the fibrillization kinetics of Aβ42 by thioflavin (ThT) assay which showed that bovine AA amyloid robustly cross-seeds human Aβ42 fibrillization in vitro (Fig. 3A–D). Next, Aβ40 was tested in the same assay which likewise resulted in a shorter lag phase in the presence of bovine AA amyloid seeds (Fig. 3E,F), indicating that AA amyloid isolated from cattle possesses the ability to cross-seed also the less aggregation-prone Aβ40. These observations show that bovine AA amyloid efficiently cross-seeds human Aβ to form amyloid fibrils in vitro.

Cross-seeding between unrelated proteins has been shown in vivo for the murine AA model^13,14^. Cross-seeding between cerebral amyloid proteins has been shown in vitro^15,16^ as well as between such proteins and non-human amyloid fibrils, e.g. originating from the gastrointestinal microbiome^17,18^. Consequently, the possible importance of this mechanism for the development of amyloid-associated dementias is extensively discussed. To the putative initiators of Aβ aggregation we now add bovine AA amyloid.

In conclusion, we show that bovine tissues used for human consumption may contain AA amyloid. The implications of this on human health are potentially far-reaching; several animal studies have shown that AA amyloidosis can spread via oral ingestion of AA fibrils and here we show that bovine AA fibrils seed human Aβ. Our findings highlight the importance of further studies to determine the biohazard of ingestion of amyloid-containing food products.

## Methods

Tissue samples were obtained from 97 cattle from a Swedish slaughterhouse, and from 99 cattle from an Italian slaughterhouse, all aged 4 years and above. All animals were rated as healthy and slaughtered for meat production. Samples from both kidneys and striated muscle (diaphragm) were fixed in 4% neutral buffered formaldehyde. Tissue samples were embedded in paraffin, and sections were stained with alkaline Congo red solution and examined in a polarization microscope. Sections from appropriate blocks with amyloid were immunolabelled with a rabbit antiserum against mouse protein AA^19^, which cross-reacts immunohistochemically with protein AA from multiple mammalian species, including cattle.

Formalin-fixed paraffin-embedded spleen tissue from four Italian cattle with AA amyloidosis and frozen Swedish beef liver was used for DNA extraction. Sequencing of exons 2, 3 and 4 followed standard procedures, details given in Supplementary information. Cross-reaction of anti Aβ mab 4G8 with human protein AA was studied in Western blot and slot blot analyses as given in Supplementary information.

Bovine protein AA, purified from fibrils from glomeruli of an animal with heavy amyloidosis^20^ were reconstituted to fibrils at 10 mg/ml in concentrated acetic acid and diluted to 1 mg/ml (0.1 mM) with 0.1 mM PBS buffer followed by continuous shaking for 7 days (Intelli-Mixer RM-2L (ELMI Ltd, Riga, Latvia). The presence of fibrils was confirmed by staining with Congo red followed by an examination in a polarization microscope and by transmission electron microscopy after negative staining with 2% uranyl acetate in 50% ethanol, imaged using a JEOL JEM2100F field emission gun transmission electron microscope (JEOL, Japan) operating at 200 kV. The reconstituted fibrils appeared irregular and curvy (Fig. 1G), different from native amyloid fibrils but similar to what was found with reconstituted fibrils from purified human protein AA^21^.

Recombinant human MetAβ42 and MetAβ40, here called Aβ42 and Aβ40, were produced as described^22^. Briefly, the crude Aβ42 or Aβ40 proteins were lyophilized overnight and re-dissolved in 7 M guanidine-HCl and then injected onto a Superdex 75 column (GE Healthcare, UK) for monomer isolation in 20 mM sodium phosphate pH 8.0 (for Aβ42) or pH 7.2 (for Aβ40) with 0.2 mM EDTA and 0.02% NaN_3_. For analysis of the kinetics of amyloid fibril formation, 20 µL solution containing Aβ monomers (1.6, 3 and 5 µM for Aβ42, 5 and 10 µM for Aβ40), 10 µM Thioflavin T (ThT) and AA amyloid (400 × dilution from the stock giving a final concentration of 250 nM (calculated as protein AA monomeric concentration) were added to each well in quadruplicate of half-area 96-well black polystyrene microplates with clear bottom and nonbinding surface (No. 3766, Corning, USA), and incubated under quiescent conditions at 37 °C. The fluorescence was recorded using a 440 nm excitation filter and a 480 nm emission filter (FLUOStar Galaxy from BMG Labtech, Offenberg, Germany).

Fischer’s exact test was used for comparison of dichotomous values. Continuous values are given as mean ± SD, and differences were evaluated by Mann–Whitney test. A *P*-value < 0.05 was regarded as statistically significant.

## Supplementary Information

Supplementary Information.
