# Supplementary material for: AA amyloid in human food chain is a possible biohazard
Source: Sci Rep. 2021 Oct 26;11:21069. doi: 10.1038/s41598-021-00588-w (PMC8548551; doi:10.1038/s41598-021-00588-w)
Supplement: Supplementary file 1 — Supplementary Information. [file 41598_2021_588_MOESM1_ESM.docx]

**Supplementary Information**

**DNA sequencing**

FFPE DNA Purification Kit K0882, and GeneJET Genomic DNA Purification Kit, K0722 were from Thermo Fisher Scientific, Uppsala, Sweden). Exons 2, 3, and 4 of the SAA gene were amplified (Table S1) with PCR performed by denaturation at 95˚C for 5 min and 40 cycles of 95˚C for 1 minute, 50˚C for 1 minute, and 72˚C for 1 minute. PCR products were purified (geneJET PCR Purification Kit K0701) and sequenced (Eurofins, Ebersberg, Germany).

**Cross-reaction of anti Aβ Mab 4G8 with human protein AA**

The anti Aβ mouse monoclonal antibody (mab) 4G8 (against Aβ 17-24 (LVFFAEDV)) was obtained from Sigma-Aldrich (Stockholm, Sweden), and rabbit antiserum A126 raised against human SAA residues 24-34 (MREANYIGSDK) has been described earlier^1^. For mapping of the epitope in human SAA recognized by mab 4G8, we used common AA amyloid with a major species 1-76 of SAA and vascular AA amyloid with two AA peptides corresponding to 2-45 and 2-95 of SAA^2,3^. Sections from formalin-fixed, paraffin-embedded kidney samples were deparaffinized and extracted with 70% formic acid. The supernatants were evaporated under N_2_, and the pellets were solubilized in SDS-PAGE sample buffer and then subjected SDS-PAGE and Western blot.

**Bovine protein AA fibrils**

Bovine protein AA, purified from fibrils from glomeruli of a cow with heavy amyloidosis, was available at the laboratory^5^ and was used in the seeding studies. Amyloid fibrils were reconstituted by solubilization of protein AA 10mg/ml in concentrated acetic acid and dilution to 1 mg/ml (0.1mM) with 0.1 mM PBS buffer followed by continuous shaking for 7 days (Intelli-Mixer RM-2L ELMI Ltd, Riga, Latvia). The presence of fibrils was confirmed by staining of a drop dried on a glass slide with Congo red followed by an examination in a polarization microscope and by transmission electron microscopy after negative staining with 2% uranyl acetate in 50% ethanol.

**Supplementary references**

1. Westermark, P., Davey, E., Lindbom, K. & Enqvist, S. Subcutaneous fat tissue for diagnosis and studies of systemic amyloidosis. *Acta Histochem.* **108**, 209-213 (2006).

2. Westermark, G.T., Westermark, P. & Sletten, K. Amyloid fibril protein AA. Characterization of uncommon subspecies from a patient with rheumatoid arthritis. *Lab. Invest.* **57**, 57-64 (1987).

3. Westermark, G.T., Sletten, K. & Westermark, P. Massive vascular AA-amyloidosis: A histologically and biochemically distinctive subtype of reactive systemic amyloidosis. *Scand. J. Immunol.* **30**, 605-613 (1989).

4. Oskarsson, M.E.*, et al.* In vivo seeding and cross-seeding of localized amyloidosis: A molecular link between type 2 diabetes and Alzheimer’s disease. *Am. J. Pathol.* **185**, 834-846 (2015).

5. Westermark, P., Johnson, K.H., Westermark, G.T., Sletten, K. & Hayden, D.W. Bovine amyloid protein AA: isolation and amino acid sequence analysis. *Comp. Biochem. Physiol. B* **85**, 609-614 (1986).

**Table S1**

Primers used for sequencing of Bos SAA1 including the signal peptide

**Forward primer (5’-3’) Reverse primer (5’-3’)**

**Exon 2** GCTCTCCTCGACATACAAAC TAGAGGCCCAAGAATGCCAT

**Exon 3** CTCCCTCTGATAATCCTTACTCC TAAGCAGCCAAGCTCTGCTCA

**Exon 4** GATCCCTTGGAAAGAGGAGA AGAACTCTCTAGGTCAGCGT

**Supplementary Fig. 1**


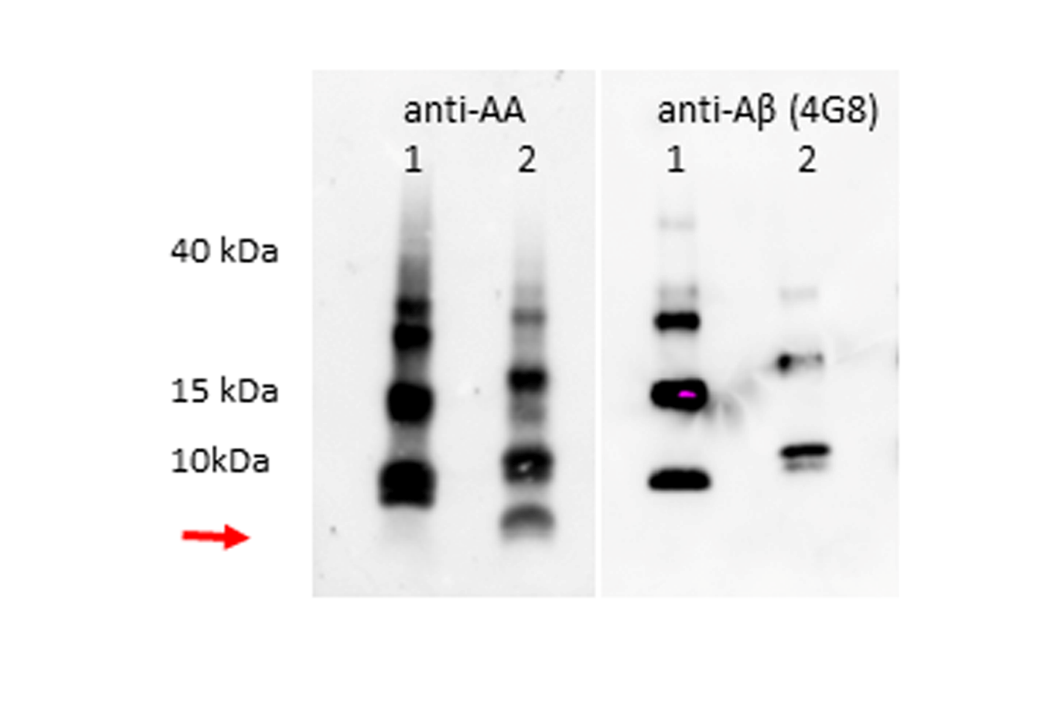


Western blot of human common AA amyloid (1) with a major species 1-76 of SAA and vascular AA amyloid with two AA fragments corresponding to 2-42 and 2-93 (2), Rabbit anti-human AA antibody raised against SAA residues 24-34 (MREANYIGSDK). The AA antibody reacts with all protein AA bands present. The right panel shows that the anti Aβ antibody 4G8 labels the same AA bands except that with the lowest molecular weight, which is labelled with antibodies against protein AA (red arrow).
